# How a Bill Becomes a Law, or How a Truly Terrible Bill Becomes Less Awful

**DOI:** 10.5811/westjem.2019.5.43666

**Published:** 2019-07-01

**Authors:** Aimee Moulin, Elena Lopez-Gusman

**Affiliations:** * University of California, Davis, Department of Emergency Medicine, Sacramento, California; † California American College of Emergency Physicians, Sacramento, California

It started slowly. On February 12, 2013, James Flavy Coy Brown arrived in downtown Sacramento after being placed on a three-day Greyhound ride on discharge from a Nevada psychiatric hospital. Less than three months later the story was exposed by the *Sacramento Bee*^1^ and ultimately led to a class action lawsuit on behalf of the patients, and a Pulitzer nomination for the paper. The following year brought a lawsuit against a hospital in Los Angeles for discharging a patient to a local shelter. In late 2016, an outbreak of Hepatitis A in San Diego’s homeless population again highlighted the poor health conditions of California’s growing homeless population. The following years brought a flood of news stories highlighting the plight of California’s homeless populations, culminating in a general sense that something should be done.

On February 14, 2018, we learned what that something was. California Senate Bill (SB) 1152 was introduced by Senator Ed Hernandez, an optometrist representing the San Gabriel Valley. At the time, while he was serving the final year of his Senate term, he was still the powerful chair of the Senate Health Committee and he was running for lieutenant governor. With the support of powerful state unions, the bill proposed limits on both hospital and emergency department (ED) homeless patient discharges.

As introduced, the bill essentially prohibited discharging homeless patients from hospitals and EDs. Homeless patients could not be discharged at night, or into inclement weather. Homeless patients could only be released to a care facility or social services agency that had agreed in writing to accept that patient. Prior to discharge, homeless patients were to receive a meal, appropriate clothing, a 30-day supply of all medications, all necessary durable medical equipment, infectious disease screening, all appropriate vaccinations, a source of regular follow-up care, a psychiatric evaluation, and transportation to any place of their choosing. Remember this was not a guideline or a recommendation for best practice. There was no room for clinical decision-making or variation in practice patterns; it would be a crime not to comply. Yes, it was intended to include patients seen only in the ED.

The fundamental challenge is that our policymakers and legislators do not share our understanding or experiences. Their contact with emergency medicine (EM) is as a patient and family member, or through news stories of sympathetic patients. The concept of the Emergency Medicine Treatment and Active Labor Act (EMTALA) which is so embedded into our daily practice and fundamental to our mission as a specialty, is poorly understood by policymakers. Those of us on the frontlines inherently understood that SB 1152 would decimate California EDs’ ability to treat patients. But from the outside it looks like basic human decency, backed by the most powerful players in California politics.

California’s Chapter of the American College of Emergency Physicians (ACEP) is almost as old as ACEP itself. At 47, the California chapter has a track record of fighting for our specialty and our patients. California ACEP is the voice of EM in the California State Capitol. The chapter has invested in our state policymakers for years. The work of explaining the unique challenges of an ED and building champions has to begin long before there is a need. Relationships and trust must also be built with other stakeholders in the political process, not just with legislators. It’s the years of building relationships and a reputation as a patient advocate that gives California ACEP influence.

California ACEP’s opposition letter to SB 1152 outlined the bill’s impact on crowding and patient care in the ED. Throughout the remainder of the spring, the California Chapter continued to meet with legislators to educate them on the impact on our ED patients. The first stop for SB 1152 was the Senate Health Committee chaired by its author Senator Hernandez.

We relied on the background work educating legislators that happens every year when our members go to Sacramento for lobby day and take policymakers on ED tours in their communities. We also worked with the sponsors of the bill to help them understand the unintended consequences of their proposal and to make changes to the bill.

Lobbying against a bill always begins with the author and their staff in the hopes that, if you can provide a better understanding of the policy and its potential impact, they will be willing to make modifications. If that doesn’t work, or they aren’t willing to make enough changes, the next step is to lobby the committee chair and the committee consultant – the staff person assigned to analyze the bill. The chair of each committee has tremendous power to reshape legislation that is heard in his or her committee. And while each committee has many members, they often defer to the chair, and they are certainly reluctant to oppose the chair. Unfortunately for us, in this instance the chair was also the author, so we weren’t going to be able to rely on the committee making changes for us. We lobbied each of the nine members of the committee, and many of them raised questions and gave voice to our concerns during the committee hearing. However, they ultimately voted for the bill. It passed out of committee with all seven Democrats voting in favor, one Republican voting no, and the other Republican abstaining.

The Chapter reached out to the California Medical Association, the California Hospital Association, and our public hospital partners to keep up pressure on our state legislators to negotiate the provisions of the bill. Throughout this process there was a continual back-and-forth conversation of potential changes and amendments. California ACEP worked hard to get to a place that we felt could provide for the needs of the homeless population, while allowing EDs the space and resources to continue to provide emergency care.

Usually bills that have a potential cost to the state are referred to the Appropriations Committee in each house for a fiscal analysis. Costs to the state are estimated for each bill, and those with a cost of more than $150,000 are placed on the “suspense file” to be considered at the end of the fiscal committee deadline. This is meant to be a thoughtful, deliberative process to maintain fiscal accountability, while various new programs/initiatives are considered each year. However, this process is often also used as a political tool to kill a bill without voting it down. It is not a stretch to estimate SB 1152 would increase costs to the state through the Medi-Cal program and increase costs to public and University of California hospitals. However, with a senator as its author and powerful political winds behind SB 1152, it bypassed the Senate Appropriations Committee process entirely and went straight to the Senate floor to be voted on by all senators.

It passed out of the Senate on a straight party line vote: all 26 Democrats voted in favor and all 13 Republicans voted against. After passing the California Senate a bill goes through a mirror process in the California Assembly before going to the Governor. The Assembly gave us another opportunity to express our concerns with lawmakers and seek amendments. Since we had a more objective committee chair in the Assembly, and because the bill was sent to the Appropriations Committee in the Assembly, there were more opportunities for our lobbying to be fruitful. It was in this process that we were able to impact the outcome of the bill.

As a result of California ACEP’s work, six sets of amendments were made to SB 1152, each lessening the impact on care provided to all patients in the ED. For example, homeless patients could be discharged when clinically appropriate, and the rest of the bill’s mandates could take place in an area of the hospital that does not provide clinical care. Homeless patients could be given transportation to a place of their choosing, rather than only to social service providers that may or may not exist or have available capacity. On August 28, SB 1152 passed out of the Senate and landed on Governor Jerry Brown’s desk.

Governor Brown was always a wild card in this debate. His passion has always been for California’s infrastructure and climate change, rather than healthcare. Also at play was Governor Brown’s style of governing. While not anti-government, he has been thoughtful and judicious when considering imposing new state requirements. While more unpredictable than most governors, he was more likely to veto legislation that places mandates on private businesses and local governments than most Democratic governors. He often said he saw the unintended consequences of the mandates he signed during his first gubernatorial terms from 1975–1983 both as a private citizen and then as mayor of Oakland.

Again, we mobilized, this time calling upon our members who sent over 700 messages to the Governor urging him to veto the bill.

Yet late in the evening on Sunday September 29, 2018, just hours before his deadline to act, Governor Brown signed SB 1152 into law. At the time it felt like a crushing defeat. However, looking back at the original bill, the efforts of California ACEP are clear. Even in defeat, I am reminded how important it is for every emergency physician to stay engaged for the health of our specialty and our patients. Recall that the original bill did not allow discharge of a homeless patient in inclement weather. Another of the many requirements was that a homeless patient be “permitted to remain in the facility for the time necessary to ensure that he or she is released during daytime hours where the receiving social services or other agency is open and available to receive the patient.” The final version of the bill requires the hospital to identify a post-discharge destination, which could include a patient’s “home.” As far as the requirements on the treating physicians before patient discharge, there were only three in the final bill, and none of them are substantially different from what we already do. They are as follows:

- The treating physician has provided a medical screening examination and evaluation. If the treating physician determines that the results of the medical screening examination and evaluation indicate that follow-up behavioral healthcare is needed, the homeless patient shall be treated or referred to an appropriate provider.
- The treating physician has determined the homeless patient’s clinical stability for discharge, including, but not limited to, an assessment as to whether the patient is alert and oriented to person, place, and time, and the physician or designee has communicated post-discharge medical needs to the homeless patient.
- The homeless patient has been provided with a prescription if needed, and for a hospital with an onsite pharmacy licensed and staffed to dispense outpatient medication and an appropriate supply of all necessary medication, if available.

Thousands of bills are introduced each year in the state legislature. In 2018 the California state legislature considered over 2,000 bills. California ACEP takes a broad look for any potential impact on our patients and our healthcare system. Each of the bills are reviewed by California ACEP staff. Several hundred bills are reviewed by California ACEP’s Government Affairs Committee and selected for either support, oppose or “watch” positions. Many bills are written poorly, and we must try to seek amendments to them to avoid unintended consequences. This process, while seemingly simple, is very resource-intensive. Additionally, California ACEP carefully watches hundreds of relevant bills during the process in case one is amended in a harmful way for our patients or practice. Well-intentioned ideas can be unworkable in the busy 24/7 pace of EM. One example is the requirement for prescribers in California to check the state Prescription Drug Monitoring Program prior to prescribing controlled substances. In recognition of our practice environment, California ACEP successfully lobbied for an exemption for prescriptions for less than seven days duration, saving untold hours of precious practice time, while protecting patients in pain.

In addition, each year, critical issues for our patients and our practice lead to chapter-sponsored bills. Currently California ACEP is sponsoring an effort to support ED patient navigators for substance use and behavioral health disorders, as well as legislation to allow emergency physicians to continue to operate as independent contractors despite a Supreme Court ruling that threatens this long-term practice. The Chapter typically sponsors four bills each year. Some take multiple attempts over several years to be enacted, while others are successful on the first try. We have sponsored at least one bill each year for the last several years to improve our ability to care for patients with mental illness. While that has been a consistent theme, our sponsored legislation has covered a wide variety of practice topics. For example, we sponsored and successfully enacted legislation that allows health information technology such as the Emergency Department Information Exchange to access information from the CURES (Controlled Substance Utilization Review and Evaluation System) database. Prior to our bill, this was prohibited by California law.

While we do not have a perfect track record, our record defeating, fixing, supporting, and sponsoring legislation is stellar. This is even more true when you consider the resources available to us. In 2017, the *Sacramento Bee* published a list of the top 500 lobbyist employer spenders. The California Hospital Association ranked sixth, the California Medical Association ranked 19^th^, the Service Employees International Union (the sponsors of SB 1152) ranked third, and California ACEP ranked 215^th^. Much like the emergency physicians we represent, California ACEP is adept at doing more with less and producing impressive outcomes. We owe much of it to the passionate voices of our members working across the state. We hope you will join us and add your voice to the fight.
